# Genetic evidence for subspecies differentiation of the Himalayan marmot, *Marmota himalayana*, in the Qinghai-Tibet Plateau

**DOI:** 10.1371/journal.pone.0183375

**Published:** 2017-08-15

**Authors:** Jingyan Yan, Hongjian Chen, Gonghua Lin, Qian Li, Jiarui Chen, Wen Qin, Jianping Su, Tongzuo Zhang

**Affiliations:** 1 Key Laboratory of Adaptation and Evolution of Plateau Biota, Northwest Institute of Plateau Biology, Chinese Academy of Sciences, Xining, Qinghai, China; 2 Qinghai Key Laboratory of Animal Ecological Genomics, Xining, Qinghai, China; 3 University of Chinese Academy of Sciences, Beijing, China; 4 Qinghai Institute for Endemic Disease Prevention and Control, Xining, Qinghai, China; National Cheng Kung University, TAIWAN

## Abstract

The primary host of plague in the Qinghai-Tibet Plateau (QTP), China, is *Marmota himalayana*, which plays an essential role in the maintenance, transmission, and prevalence of plague. To achieve a more clear insight into the differentiation of *M*. *himalayana*, complete cytochrome *b* (cyt *b*) gene and 11 microsatellite loci were analyzed for a total of 423 individuals from 43 localities in the northeast of the QTP. Phylogenetic analyses with maximum likelihood and Bayesian inference methods showed that all derived haplotypes diverged into two primary well-supported monophyletic lineages, I and II, which corresponded to the referential sequences of two recognized subspecies, *M*. *h*. *himalayana* and *M*. *h*. *robusta*, respectively. The divergence between the two lineages was estimated to be at about 1.03 million years ago, nearly synchronously with the divergence between *M*. *baibacina* and *M*. *kastschenkoi* and much earlier than that between *M*. *vancouverensis* and *M*. *caligata*. Genetic structure analyses based on the microsatellite dataset detected significant admixture between the two lineages in the mixed region, which verified the intraspecies level of the differentiation between the two lineages. Our results for the first time demonstrated the coexistence of *M*. *h*. *himalayana* and *M*. *h*. *robusta*, and also, determined the distribution range of the two subspecies in the northeast of QTP. We provided fundamental information for more effective plague control in the QTP.

## Introduction

The plague, historically known as the “Black Death” because of the 14^th^ century pandemic, is an infectious disease caused by the bacterium *Yersinia Pestis*. The massive pandemics of the plague in Eurasia resulted in the death of an estimated 75 to 200 million people and caused significant economic, cultural, and population changes worldwide [1]. It remains a lethal threat to human society nowadays, and sporadic plague outbreaks still happen in many parts of the world [2]. According to the World Health Organization, there were 3248 cases reported worldwide with 584 deaths from 2010 to 2015 (http://www.who.int/mediacentre/factsheets/fs267/en/).

Many stable enzootic plague foci on every major inhabited continent except Australia have come into being, among which China makes up a great part [2]. In China, the natural focus of Himalayan marmot plague on the Qinghai-Tibet Plateau (QTP), with an area of 630 000 km^2^, is the largest and most active one [3]. Human deaths caused by plague in this focus were reported even in recent years [4]. As the most important enzootic host in this focus, the Himalayan marmot (*Marmota himalayana*), plays a key role in the maintenance, transmission, and prevalence of plague, and is directly or indirectly responsible for most human epidemics [4]. Therefore, studies on biological features of Himalayan marmot are greatly beneficial for the prevention and control of plague. However, previous studies on this animal are mainly restricted to simple population investigations (e.g. population distribution, flea infestation, plague prevalence, etc.), while very few studies extend to other areas such as the molecular biology [5, 6].

Marmots (family Sciuridae, genus *Marmota*) are large squirrels and widespread in the Holarctic [7]. Four marmot species, *M*. *baibacina* (Kastschenko, 1899), *M*. *sibirica* (Radde, 1862), *M*. *caudata* (Geoffroy, 1844) and *M*. *himalayana* (Hodgson, 1841), have been found in China [8]. Among those, *M*. *himalayana*, endemic to the QTP, is the most abundant and widely distributed one. The taxonomic status of *M*. *himalayana* as an independent species closest to *M*. *sibirica* and *M*. *camtschatica* is well evidenced and widely accepted [9, 10]. However, as to the more exquisite phylogenetic relationship and more recent differentiation in this species, few literatures can offer any knowledge about it. After *M*. *himalayana*, *Arctomys himalayanus* (Hodgson, 1841), and *M*. *robusta*, *Arctomys robustus* (Milne-Edwards, 1871), were successively put forward, the relationship between them, or the taxonomic status of the latter, is controversial and obscure up to now. Based on a simple morphological comparison between specimens of the two types, De Winton and Styan (1899) [11] regarded them as identical while Jacobi (1923) [12] and Howell (1929) [13] considered the named *M*. *robusta* ‘merely a subspecies of the more western *M*. *himalayana*’ and described their diagnostic characters as follows: ‘(*M*. *h*. *himalayana*) apparently differs in being grayer, without the more ochraceous tints of *M*. *h*. *robusta*, and in having the black of the forehead less extensive’. Although Allen (1938) [14] inclined to agree with De and Styan (1899), considering the paucity of specimens available, he just retained it. After these, discussions about this issue can still only be accessed in simple checklists, most of which stood for the subspecies idea but reached few consensuses as to the distribution of two types and conveyed no information about their distinct characteristics and differentiation [15, 16].

It is evident that additional studies are needed to figure out the genetic diversity and intraspecific differentiation in *M*. *himalayana* and to clarify the relationship between *M*. *h*. *himalayana* and *M*. *h*. *robusta*. Here, we used the complete mitochondrial cytochrome *b* gene (cyt *b*), and 11 microsatellite markers to investigate the genetic diversity and differentiation of the marmots in northeastern QTP. We tried to answer the following questions: 1) are the two independent taxonomic units, the named *M*. *h*. *himalayana* and *M*. *h*. *robusta*, both frequently distributed in northeastern QTP? 2) If yes, where are they respectively distributed and what’s their taxonomic status and relationship?

## Materials and methods

To enhance the reproducibility of our results, the laboratory protocol was deposited in protocols.io (http://dx.doi.org/10.17504/protocols.io.iyxcfxn). Any possible questions about the step-by-step protocol can be discussed with the authors on this site.

### Sample collection and DNA extraction

**Fig 1:**
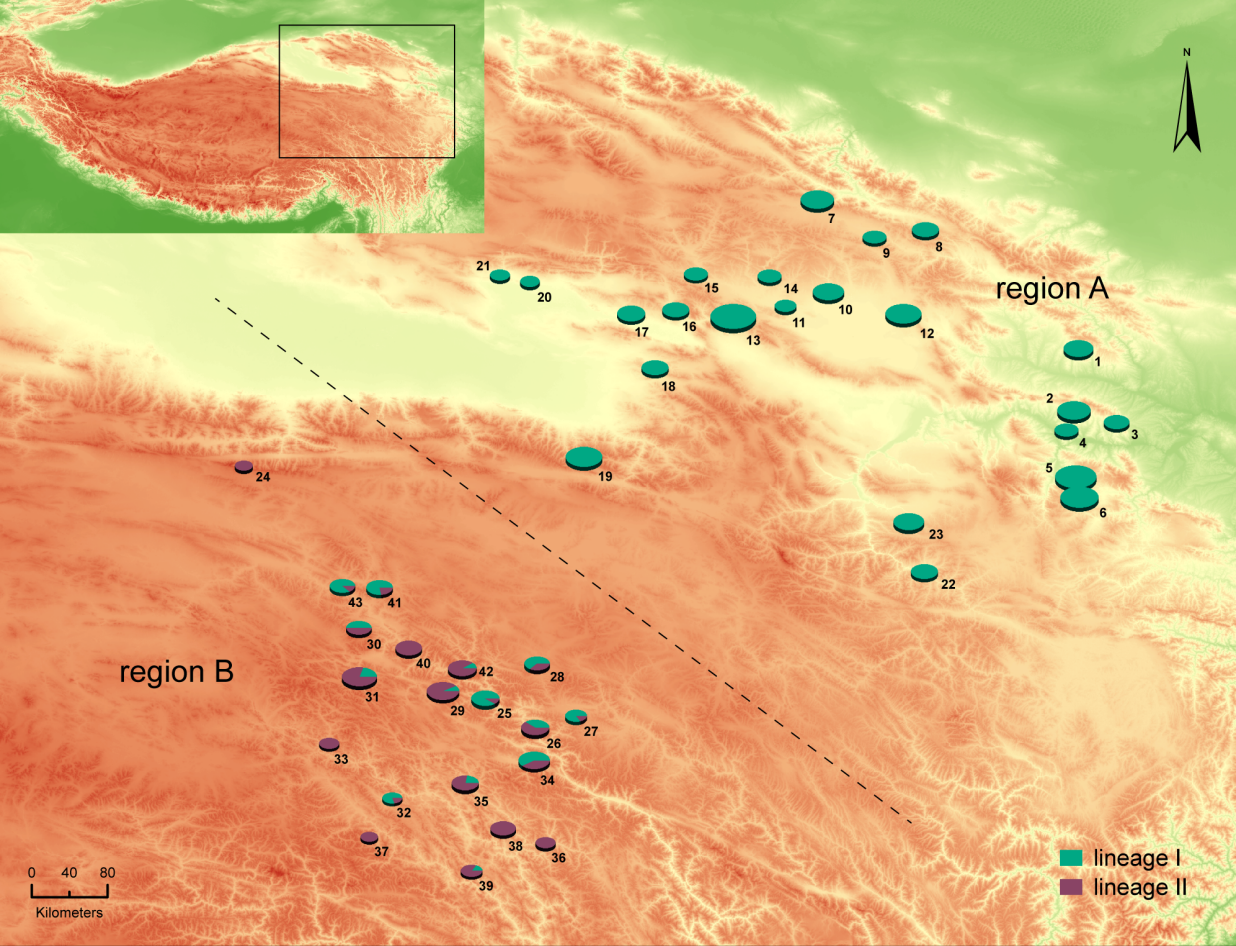
Geographical distribution and haplotype frequencies of 43 populations of *M*. *himalayana* in this study. The size of pie charts indicates the sample size of each population with the blue green part corresponding to the percentage of haplotypes belonging to lineage A and the purple red part the percentage of haplotypes belonging to lineage B. The dashed line divides the total area into two parts: Region A, covering populations that consist of only haplotypes in lineage I, and Region B, covering populations that include haplotypes in both lineages and several ones located among them with only haplotypes in lineage II.

423 muscle specimens were collected from 43 localities in northeastern QTP (Fig 1, Table 1 and S1 Table) during the period 2014 to 2016. For each locality, at least 4 individuals were randomly sampled. After trapped, the marmots were anaesthetized to death by 100% diethyl ether. And then, muscle tissues were removed and stored in 95% ethanol immediately. Total genomic DNA was extracted using the commercial kit (Ezup Column Animal Genomic DNA Purification Kit, Sangon Biotech).

**Table 1:** Genetic diversity of 43 populations of *M*.*himalayana*.

Pop	Code	n	*Ho*	*H*e	π (×10^−2^)	*h*	cyt *b* haplotype frequencies (haplotype ID-number)
1	HZK	11	0.73±0.25	0.69±0.20	0.28	0.62	H31-1, H32-6, H33-4
2	LDQ	14	0.58±0.20	0.60±0.19	0.06	0.27	H10-12, H11-1, H32-1
3	MHL	8	0.78±0.13	0.71±0.09	0.00	0.00	H23-8
4	HLE	7	0.69±0.26	0.66±0.20	0.05	0.29	H10-1, H23-6
5	XHG	21	0.79±0.14	0.74±0.11	0.10	0.69	H10-11, H59-4, H63-2, H64-1, H65-3
6	TRG	18	0.74±0.15	0.79±0.07	0.10	0.72	H10-9, H17-1, H51-4, H57-2, H58-1, H59-1
7	QLZ	14	0.75±0.13	0.72±0.09	0.22	0.66	H17-1, H37-8, H38-3, H39-1, H40-1
8	QLE	9	0.66±0.21	0.60±0.16	0.00	0.00	H17-9
9	QLM	7	0.82±0.21	0.74±0.10	0.00	0.00	H17-7
10	GCY	12	0.76±0.14	0.74±0.09	0.15	0.79	H10-2, H17-5, H18-3, H21-1, H22-1
11	GCJ	6	0.74±0.20	0.73±0.16	0.06	0.60	H10-1, H17-4, H20-1
12	HYG	16	0.68±0.16	0.70±0.15	0.17	0.65	H11-3, H28-9, H29-3, H30-1
13	TJX	26	0.74±0.08	0.76±0.06	0.01	0.15	H10-2, H17-24
14	TJZ	7	0.71±0.30	0.69±0.17	0.00	0.00	H17-7
15	TJY	7	0.69±0.25	0.68±0.16	0.00	0.00	H17-7
16	WLN	9	0.74±0.22	0.70±0.17	0.02	0.22	H17-8, H37-1
17	WLY	10	0.71±0.16	0.66±0.12	0.08	0.73	H17-5, H37-2, H61-2, H62-1
18	WLW	9	0.87±0.13	0.74±0.08	0.02	0.22	H17-8, H60-1
19	DLQ	17	0.69±0.21	0.68±0.13	0.10	0.58	H07-7, H17-9, H19-1
20	DHK	5	0.58±0.24	0.54±0.21	0.37	0.80	H15-2, H16-1, H18-2
21	DHH	5	0.73±0.29	0.63±0.20	0.37	0.80	H15-2, H16-2, H17-1
22	TDH	9	0.64±0.16	0.76±0.11	0.12	0.86	H10-2, H17-2, H51-3, H54-1, H56-1
23	TDG	12	0.79±0.12	0.80±0.09	0.15	0.88	H10-2, H50-2, H51-4, H52-1, H53-1, H54-1, H55-1
24	GEX	4	0.52±0.21	0.55±0.16	0.00	0.00	H13-4
25	CDG	10	0.75±0.19	0.75±0.14	0.57	0.80	H02-1, H08-1, H09-3, H10-4, H11-1
26	CDC	10	0.78±0.18	0.72±0.16	1.37	0.62	H05-6, H06-2, H07-2
27	CDZ	6	0.80±0.15	0.78±0.13	0.91	0.80	H10-1, H12-1, H13-1, H14-3
28	CDQ	8	0.76±0.20	0.71±0.15	1.03	0.90	H07-3, H12-2, H13-3
29	ZDL	13	0.75±0.17	0.72±0.12	0.49	0.83	H02-4, H08-1, H24-3, H25-3, H26-2
30	ZDZ	8	0.78±0.19	0.69±0.14	1.46	0.75	H13-1, H47-4, H48-1, H27-2
31	ZDD	15	0.71±0.19	0.70±0.18	1.13	0.70	H03-2, H10-1, H48-8, H49-2, H24-2
32	ADA	5	0.68±0.27	0.68±0.18	0.95	0.40	H01-4, H02-1
33	ADZ	5	0.65±0.24	0.69±0.23	0.14	0.40	H03-4, H04-1
34	YSZ	12	0.75±0.23	0.74±0.18	1.21	0.68	H02-4, H10-6, H17-1, H34-1
35	YSS	9	0.79±0.29	0.67±0.21	0.99	0.78	H02-2, H04-4, H10-2, H34-1
36	YSM	5	0.69±0.23	0.66±0.18	0.00	0.00	H66-5
37	NQD	4	0.83±0.21	0.72±0.14	0.00	0.00	H35-4
38	NQM	8	0.68±0.16	0.70±0.11	0.15	0.25	H34-7, H36-1
39	NQB	6	0.65±0.25	0.61±0.17	0.91	0.60	H04-1, H17-1, H34-4
40	QMD	9	0.83±0.23	0.71±0.13	0.34	0.39	H43-2, H44-7
41	QMQ	9	0.89±0.11	0.77±0.05	1.15	0.81	H07-2, H42-4, H43-1, H45-1, H46-1
42	QMB	10	0.78±0.28	0.74±0.15	0.52	0.64	H02-4, H41-5, H42-1
43	QMY	8	0.78±0.15	0.73±0.11	0.84	0.79	H42-3, H47-3, H48-1, H49-1

Sample size (n), mean Nei’s (1978) observed (*Ho*) and expected heterozygosity (*He*), nucleotide diversity (π), haplotype diversity (*h*) and cyt *b* haplotype frequencies(haplotype ID-number) are demonstrated.

### Ethics statement

Our study was performed in strict accordance with the recommendations in the Guide for the Care and Use of Laboratory Animals of the National Institutes of Health. The protocol was approved by the Committee on the Ethics of Animal Experiments of the Northwest Institute of Plateau Biology, Chinese Academy of Sciences (Permit Number: NWIPB-2014-023). The field work was permitted by the Wild Animals and Plants and Natural Reserves Administration, Qinghai Forestry Bureau. All samples were collected by cooperators from the Qinghai Institute for Endemic Disease Prevention and Control in their routine marmot-killing tasks in order to minimize the effect of sampling on wild marmot populations and eliminate the risk of plague infection.

### Mitochondrial DNA amplification and sequencing

The complete sequence (1140 bp) of the cyt *b* gene and partial flanking segments (partial ND6 and tRNA-Glu for 5’-end, partial tRNA-Pro and tRNA-Thr for 3’-end) were amplified and sequenced using a pair of self-designed primers: CY-F (5’-ATCCTAAGCCTCCGTAAATAGGA-3’) and CY-R (5’-CAGGGAATAGTT TAAGTAAGAAATGTCA-3’) (referring to the *M*. *himalayana* mitochondrial genome under accession No. JX069958). PCRs were carried out in a 40 μL reaction volume containing 1 μL genomic DNA (approximately 60 ng), 5.0 μL of 10×PCR buffer (with Mg^2+^), 2.5 μL of dNTPs (2.5 mM), 1 μL of each primer (10 μM), 2.5 units Taq DNA polymerase (5 U/μL) and 29 μL sterilized double-distilled water. The PCR conditions used were as follows: an initial denaturation at 95°C for 5 minutes followed by 30 cycles of denaturation at 95°C for 45 seconds, annealing at 57°C for 45 seconds, and extension at 72°C for 1 minute 45 seconds, and a final extension at 72°C for 10 minutes. PCR products were then sequenced in both directions by the Shanghai Sangon Biological Engineering Technology & Services Co., Ltd (Shanghai, China).

### Microsatellite amplification and genotyping

All 423 specimens were genotyped at 11 microsatellite loci, B, D, E, G, H, I, J, M, R, T, and W [17]. PCRs were conducted using fluorescently-labelled primers in a total volume of 20 μL that contained 0.5 μL genomic DNA (approximately 30 ng), 2 μL of 10×PCR buffer (with Mg^2+^), 1 μL of dNTPs (2.5 mM), 0.4 μL of each primer (10 μM), 1 unit Taq DNA polymerase (5 U/μL) and 15.5 μL sterilized double-distilled water. The amplification profile was: an initial denaturation at 95°C for 4 min followed by 40 cycles of denaturation at 95°C for 45 sec, annealing at Tm (annealing temperature, as listed in Table 2) for 25 sec and extension at 72°C for 20 sec, and a final extension at 72°C for 2 min. Fragments were then analyzed by the Shanghai Sangon Biological Engineering Technology & Services Co., Ltd (Shanghai, China).

**Table 2:** Characteristics of 11 microsatellite loci used here: Accession number, repeat motif, primer sequence, annealing temperature(AT, °C) and allele number (AN).

Locus	Accession number	Repeat motif	Primer sequence (5'→3')		AT (°C)	AN
			Forward	Reverse		
B	JQ317689	(GT)_16_	TTTTTGGCTAACATAGTGGT	AGTGAAGGCTAAAGCAGAGT	53	12
D	JQ317691	(GT)_19_	ATGGGGACAAACATGGGACT	CGGTTGCTATGGAGACTGGA	56	8
E	JQ317692	(TC)_24_	CTTGTTCAGGATTTGGCTAT	AATGTCTTGAAAATGGTGTT	52	14
G	JQ317694	(AG)_20_	ATGGCAGAGAATATAAAATGG	CTGGTGGAACTTGTTAGGAG	53	14
H	JQ317695	(GT)_14_	GGAAGACCACAGAGGAACAG	CCTTGAAGAGCAAGAGCATA	54	8
I	JQ317696	(TG)_12_	TAATATCCCCCAAAGAAGTA	TAGACCTTGCTGTGAAAAAT	48	13
J	JQ317697	(TG)_12_	ATGGGACAGAACTCTTGATT	CCTTATAGTTTTACCTCCTCC	56	13
M	JQ317700	(AC)_22_	CATTGGAAGACAGAAAATACA	CAGTCCTTTGAAACTTGAGTA	48	14
R	JQ317704	(AC)_11_	ACAAAACTTCTTCGTCTC	GTCTTCCACTACTCCTCT	50	7
T	JQ317706	(TG)_11_	AATAGCCAGTTCAACCTC	ATGCTAACTTCAGCAACA	53	11
W	JQ317709	(CA)_14_	TTTCCACAGCAGCACTCT	GGTTCCTTACCCAGACCA	55	10

### Phylogenetic analysis

DNA sequences were checked and aligned using MEGA v7.0.18 [18]. Polymorphic sites and haplotypes were identified using DnaSP v5.10.01 [19], with which the genetic diversity indices, haplotype diversity (*h*) and nucleotide diversity (π), were also calculated for every population. Phylogenetic relationship of the detected haplotypes was reconstructed by maximum likelihood (ML) and Bayesian inference (BI) methods. Jmodeltest v2.1.6 [20] and the Akaike Information Criterion (AIC) were used to select a best-fit DNA substitution model. Maximum-likelihood analyses were conducted in PhyML v3.0 [21] using the BEST search method. The branch support values of the ML trees were estimated using non-parametric bootstrap (BSP) with 1000 replicates. The Bayesian analyses were performed using MrBayes v3.2.3 [22]. Two simultaneous Markov Chain Monte Carlo (MCMC) analyses were run for 10 million generations, and trees were sampled every 100 generations. The first 25% of trees were discarded as ‘burn-in’, and the remaining samples were used to generate the consensus tree and the Bayesian posterior probability (BPP). Phylogenetic trees were visualized with Figtree v1.4.3 [23]. The consistency of the two methods (BI and ML) was evaluated through the similarity of topology and comparison of support values for identical nodes in the resultant phylogenetic trees. To indicate the phylogenetic features more clearly, another two cyt *b* sequences with confirmed taxonomic identities from Genbank, one for *M*. *h*. *himalayana* (from the genome under Accession No. JX069958) and the other for *M*. *h*. *robusta* (Accession No. AF143928), were added to the final data set, as well as a homogenous sequence for *M*. *sibirica* (Accession No. AF143937) as an outgroup to root the phylogenetic trees.

Besides, a median-joining haplotype network was calculated based on the maximum parsimony criterion with NETWORK v5.0 [24, 25] since this approach was suggested to be more efficient than classical phylogenetic methods for representing intraspecific evolution [26]. Ambiguous connections were removed to show the most definite connections clearly under the criteria described in Posada and Crandall (2001) [26].

### Genetic distance calculation

In the phylogenetic analyses, all cyt *b* haplotypes were clustered into two well-supported basal lineages, lineage I (the *himalayana* lineage) and lineage II (the *robusta* lineage). To examine the divergence degree of the two lineages, we calculated their ML genetic distance in MEGA v7.0.18 and compared it with those between some other species or subspecies pairs in marmot genus based on same sequences in Steppan *et al*. (1999) [9]. Relevant parameters were set up referring to Steppan *et al*. (1999) [9].

### Divergence time estimation

Divergence times of the well-supported basal nodes were then estimated using a strict molecular clock method in BEAST v1.8.3 [27], with a constant-size coalescent tree prior and HKY + I substitution model. Three calibrations were used. The 1st was a fossil calibration regarding *Marmota minor* as the oldest marmot fossil at 10.3 mya (J. Alroy, Macquarie University, Sydney, pers. comm.; Paleobiology Database, http://www.paleodb.org, accessed 1 January 2010). Following the protocol of Steppan *et al*. (2011) [28], a prior normal distribution with a mean of 10.3 mya and a standard deviation (SD) of 0.9 mya was assigned to the most recent common ancestor (MRCA) of marmots and their sister group, the clade of *Callospermophilus lateralis*, *C*. *saturatus*, and *Otospermophilus beecheyi*. Another two calibrations were based on estimates of Steppan *et al*. (2011) [28]. One was a normal distribution with a mean of 6.0 mya and an SD of 0.8 mya for the MRCA of subgenera *Petromarmota* and *Marmota*, and another was a normal distribution with a mean of 4.9 mya and an SD of 0.6 mya for the MRCA of subgenus *Marmota*. For the calibrations, cyt *b* sequences of another 16 relative species, *M*. *camtschatica* (AF100715), *M*. *baibacina* (AF143915), *M*. *kastschenkoi* (AF143914), *M*. *bobak* (AF143917), *M*. *menzbieri* (AF143931), *M*. *caudata* (AF143925), *M*. *marmota* (AF143929), *M*. *broweri* (AF143919), *M*. *monax* (AF143934), *M*. *olympus* (JF313271), *M*. *vancouverensis* (AF143939), *M*. *caligata* (AF143920), *M*. *flaviventris* (AF143927), *C*. *lateralis* (AF157887), *C*. *saturatus* (AF157917) and *O*. *Beecheyi* (AF157919), were added to the data set.

The MCMC was run for 10 million generations with parameters sampled every 1000 generations. With the first 10% samples discarded as burn-in, the convergence of the stationary distribution and stability of estimated parameters were examined using TRACER v1.6 [29] by inspection of plotted posterior estimates and the effective sample sizes of all parameters. The maximum clade credibility tree was summarized in TreeAnnotator v1.8.3 [30]. A tree with ages for main nodes and their 95% highest posterior density intervals was displayed using FigTree v1.4.3 [23].

### Test for reproductive isolation using microsatellite data

First, for each population, mean values across all loci for the observed heterozygosity (*Ho*) and expected heterozygosity (*He*) (Nei, 1978) were calculated using Arlequin v3.5 [31]. Departure from Hardy-Weinberg equilibrium of each population across all loci and linkage disequilibrium (LD) of all pairs of loci across populations were tested by Fisher’s exact tests using a Markov chain Monte Carlo approach with 100 000 steps and 1 000 iterations in GenePop v4.6 [32].

To verify the taxonomic level (interspecies or intraspecies) of the differentiation between the two basal lineages in the derived phylogenetic trees, i.e., the degree of the reproductive isolation between the two lineages, the most direct method should be laboratory crosses. But subjected to the limit of practice, here, we tried to find some hints from genetic structure analyses. It can be deduced that under the hypothesis of reproductive isolation of species level, strong genetic structure exactly corresponding to the species differentiation should be inferred from the rapidly varying nuclear microsatellite markers, even in the mixed range of two lineages, and of course, if the exploration for such a structure just fails, the hypothesis can definitely be rejected.

Apparently, the phylogenetic relationship and the haplotype distribution pattern (Fig 1) demonstrated that the studied area could be divided into two regions, A and B. The region A, a unique breeding range for the *himalayana* lineage, covered the populations consisting of only haplotypes in lineage I. The region B, a mixed range, covered the populations including haplotypes from both lineages and several populations located among them although with only haplotypes from lineage II. To achieve a better graphic representation and a more explicit interpretation for the differentiation between the two cyt *b*-based lineages in the genetic structure analyses, all individuals from region A were organized as group A and individuals from region B with haplotypes belonging to the *himalayana* lineage were assigned into group B-I while the rest ones with haplotypes belonging to the *robusta* lineage into group B-II. GenePop v4.6 was employed to calculate the *F*_*st*_ between three group pairs, group A vs. group B-I, group A vs. group B-II, and group B-I vs. group B-II. Then, a Bayesian model-based clustering method implemented in structure v2.3 [33] was employed to detect the genetic structure based on the microsatellite dataset. To determine the optimal number of genetic clusters (K), five independent runs were performed for each K-value ranging from 1 to 20, based on the admixture model with correlated allele frequencies. The length of the MCMCs was 500 000 steps after a burn-in period of 50 000 steps. The most likely value of K was estimated with Structure Harvester using the statistic ΔK [34, 35]. Membership coefficients were permuted using CLUMPP v1.1.2 [36]. Plots of individual assignment probabilities at the optimal value of K and several other K-values were generated with DISTRUCT v1.1 [37].

## Results

### Sequence variation and phylogenetic relationship

A 1345bp long DNA fragment covering the partial sequences of ND6 and tRNA-Pro and complete sequences of tRNA-Glu, cyt *b* and tRNA-Thr was harvested. However, only the 1140bp long sequences for the complete cyt *b* genes were used in following analyses. A total of 100 polymorphic sites, comprising 2 three-variant and 77 two-variant parsimony informative sites and 21 singleton variable sites, were detected in the aligned dataset. Among all 423 sampled individuals from 43 populations, 66 haplotypes were identified. The top two most widespread haplotypes were H17 and H10, which were respectively observed in 17 (39.5%) populations with a total frequency of 100 (23.6%) and 14 (32.6%) populations with a total frequency of 56 (13.2%). Aside from H2 found in 6 populations and 16 individuals, all the remaining haplotypes appeared in no more than 4 populations with a total frequency less than 15. Out of these, 40 were private ones, i. e., discovered in single populations.

**Fig 2:**
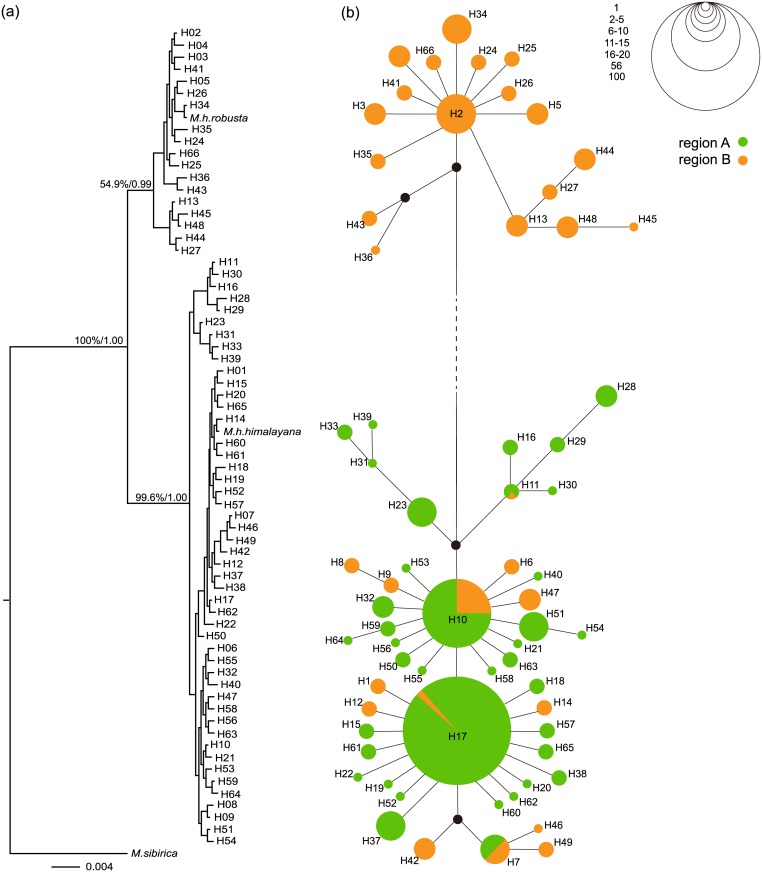
Phylogeny of the derived 66 cyt *b* haplotypes in *M*. *himalayana*. a. Bayesian phylogenetic tree with *M*. *sibirica* as an outgroup and two referential sequences representing *M*. *h*. *himalayana* and *M*. *h*. *robusta* included. BSPs (percentages) and BPPs (decimals) for main identical nodes in BI and ML trees are displayed. b. Network of the 66 cyt *b* haplotypes. Black dots indicate unsampled or extinct haplotypes. Circle sizes correspond to the haplotype frequencies with the blue part representing the percentage in Region A and the yellow part that in Region B. The line length is approximately proportional to the number of mutation steps between the connected haplotypes.

The topological structures of phylogenetic trees reconstructed by ML and Bayesian methods were consistent in the main divergences. The Bayesian tree with BSPs (percentages) and BPPs (decimals) for main identical nodes in both trees is shown in Fig 2a. All derived haplotypes have diverged into two lineages at the basal node in both trees. Two referential sequences representing *M*. *h*. *himalayana* and *M*. *h*. *robusta* were separately clustered into one of the two lineages with *M*. *h*. *himalayana* in lineage I (the *himalayana* lineage) and *M*. *h*. *robusta* in lineage II (the *robusta* lineage). The support values for this node indicated by BSP and BPP were both 1. Then, both the *himalayan* and *robustus* lineages split into two branches again. The support values were 99.6% (BSP) and 1 (BPP) for the split in the *himalayana* lineage and 54.9% (BSP) and 0.99 (BPP) for that in the other lineage. BSPs greater than 70% and BPPs greater than 0.95 were considered as strong support [38–40]. The haplotype network (Fig 2b) was greatly consistent with the Bayesian tree and confirmed again the phylogenic patterns described above.

### Genetic distance between the *himalayana* and *robusta* lineages

Our analysis showed that the genetic distance between the *himalayana* and *robusta* lineages was estimated to be 2.7% and was larger than that of any subspecies pair (0–2.2%, mean = 1.20%) listed in Steppan *et al*. (1999) [9], and even than that between *M*. *vancouverensis* and *M*. *caligata* (1.2%). Although the distance between *M*. *baibacina*. *baibacina* and *M*. *b*. *kastschenkoi* (3.5%) was larger, it was removed from the comparison since *M*. *b*. *kastschenkoi* was elevated to an independent species in Brandler (2003) [41].

### Estimates of divergence times

**Fig 3:**
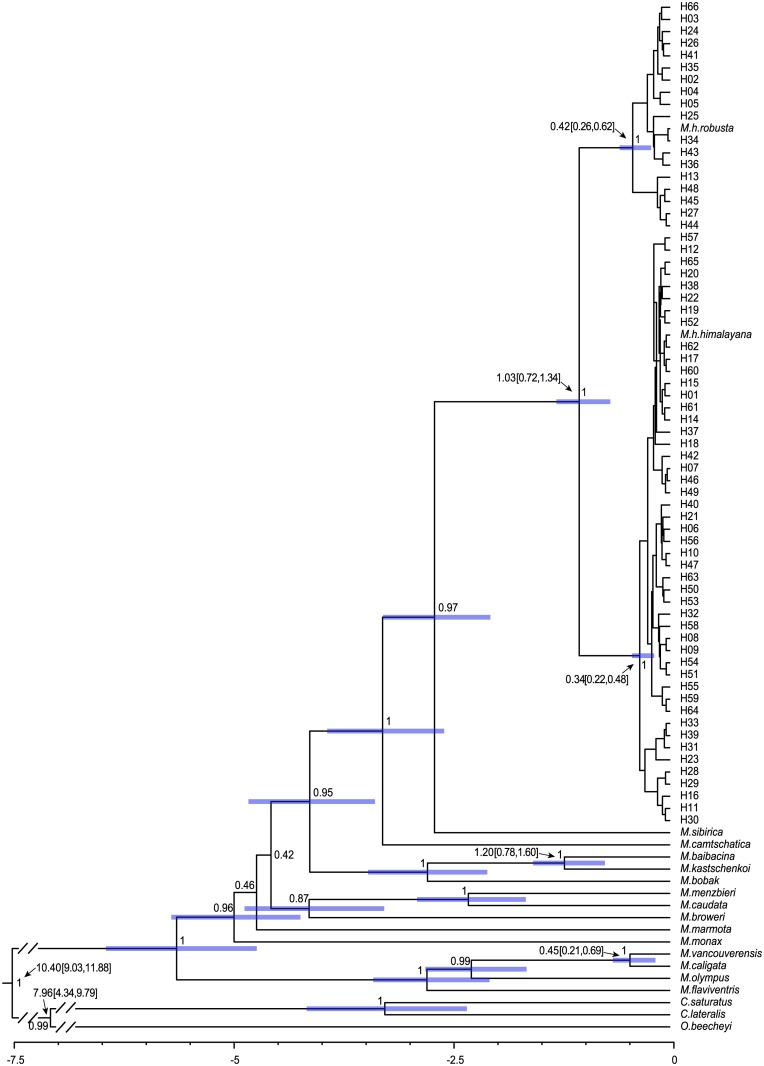
The divergence times estimated with BEAST based on cyt *b* sequence data for Marmota. Bayesian posterior probabilities for main nodes were indicated by decimal numbers around them. Mean values and 95% HPDs (in brackets) are shown behind the arrows.

The BEAST analysis (Fig 3) indicated that the divergence between the *himalayana* and *robusta* lineages occurred at about 1.03 mya (95% highest posterior density, HPD: 0.72–1.34 mya). It happened at almost the same time when the divergence between *M*. *baibacina* and *M*. *kastschenkoi* (1.20 mya; 95% HPD: 0.78–1.60 mya) took place, and it was much earlier than the divergence between *M*. *vancouverensis* and *M*. *caligata* (0.45 mya; 95% HPD: 0.21–0.69 mya). After that, the two lineages both split into two branches again, respectively at 0.34 mya (95% HPD: 0.22–0.48 mya) and 0.42 mya (95% HPD: 0.26–0.62 mya), with the latter almost simultaneously with the divergence between *M*. *vancouverensis* and *M*. *caligata*.

### Result of reproductive isolation test using microsatellite data

All 11 microsatellite loci were polymorphic. The overall number of alleles for each locus ranged from 7 (R) to 14 (E, G, M) with a total of 124 observed alleles and an average number of 11.3 (Table 2). The average genetic diversity for each population, measured as observed heterozygosity (*Ho*) and expected heterozygosity (*He*) (averaged across loci), ranged from 0.52 and 0.54 to 0.89 and 0.80 (Table 1). Only one out of all 43 populations, the number 18 population, WLW, was detected to diverge from Hardy-Weinberg equilibrium significantly (P < 0.01). Tests of linkage disequilibrium for all pairs of loci across populations yielded no significant genotypic disequilibrium (P < 0.01).

**Fig 4:**
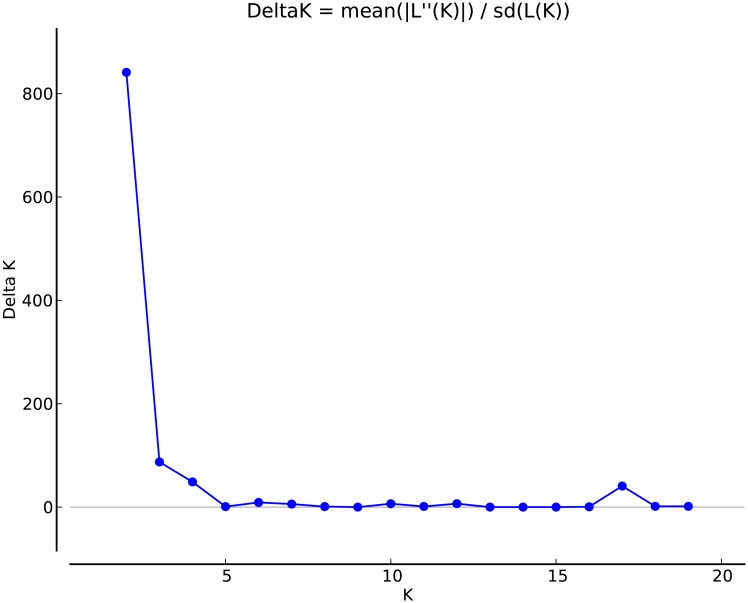
The Delta K curve generated by Structure Harvester. K = 2 was estimated as the optimal.

**Fig 5:**
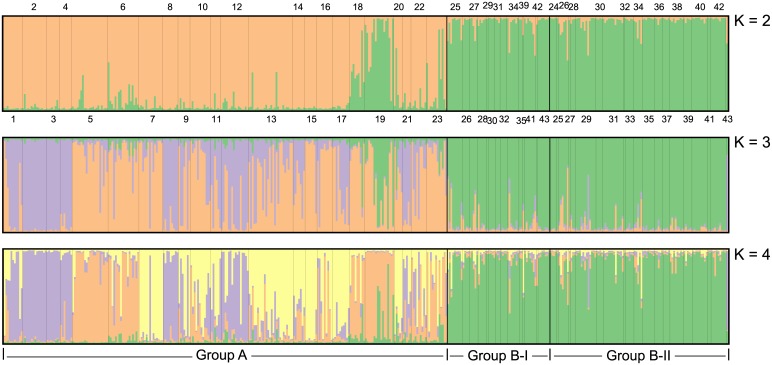
Results of Bayesian individual-based clustering in Structure with K = 2, K = 3 and K = 4. Each individual is represented by a single vertical bar divided into K colors. The colored segment shows the estimated proportion of membership to the genetic cluster.

In total, 23 populations with 259 individuals were allocated to region A, i.e. group A, while the rest 20 populations with 164 individuals were assigned to region B. In region B, 60 samples from 14 populations were divided into group B-I while the remaining 104 samples from 20 populations into group B-II. According to Structure Harvester, the optimal value for K was estimated to be 2, and then 3 and 4 (Fig 4). The bar plots of individual assignment probabilities at K = 2, K = 3 and K = 4 are shown in Fig 5. As displayed in all three situations, strong genetic differences accompanied by clear signs of admixture was detected between group A (the unique breeding range for the *himalayana* lineage) and the B-I and B-II groups (the mixed range of two lineages), while inside the mixed range, no genetic differences could be observed between the B-I and B-II groups at all, demonstrating the strong gene flow and little reproductive isolation between the two lineages in the mixed range. The meaning of the *F*_*st*_ values confirmed our results. It was estimated as 0.0677 for group A vs. group B-I and 0.0765 for group A vs. group B-II in contrast to a smaller *F*_*st*_ value for group B-I vs. group B-II (0.0067).

## Discussion

### Evidence for the *himalayana* and *robusta* lineages as independent taxonomic units

In our study, cyt *b* sequences of all 423 specimens from the northeast of QTP were clustered into two basal well-supported and monophyletic lineages, lineage I and lineage II. The lineages corresponded to the referential sequences representing *M*. *h*. *himalayana* and *M*. *h*. *robusta*, respectively (Fig 2). The genetic distance between the *himalayana* and *robusta* lineages was estimated to be 2.7%, which was larger than that of any subspecies pair (0–2.2%, mean = 1.20%) listed in Steppan *et al*. (1999) [9] and even that between two independent species, *M*. *vancouverensis* and *M*. *caligata* (1.2%). Taking the subspecies taxonomic status of the *himalayana* and *robusta* lineages we are going to illustrate here into consideration, this result seems unexpected but it is consistent with the different divergence histories. As revealed in the tMRCA analysis in BEAST (Fig 3), our two lineages were evaluated to diverge at about 1.03 mya nearly synchronously with the divergence between *M*. *baibacina* and *M*. *kastschenkoi* (1.20 mya; 95% HPD: 0.78–1.60 mya) and much earlier than that between *M*. *vancouverensis* and *M*. *caligata* (0.45 mya; 95% HPD: 0.21–0.69 mya). *M*. *vancouverensis* is a special marmot species living exclusively on an island, the Vancouver Island [42]. Although it was estimated to diverge from the continental *M*. *caligata* more recently, which can be explained by the isolation of that island from the mainland after the sea level rise at the end of the last glaciation, little chance for second contact since the isolation enabled them to evolve into different species [42]. However in *M*. *himalayana*, the situation was just the opposite due to frequent and great second contacts as discovered here in the mixed range, leaving the two diverged lineages merely developing into subspecies in *M*. *himalayana*. Back to our topic, a certain distribution pattern of haplotypes in the two lineages emerged in the studied range. Only haplotypes in the *himalayana* lineage were detected in all 259 individuals in a pretty large area in the northeast (region A). However, all haplotypes in the *robusta* lineage and a few ones in the *himalayana* lineage, specific to this region (H01, H06, H08, H09, H12, H14, H42, H46, H47 and H49) or shared with region A (H7, H10, H11 and H17), were found in the southwest (region B) (Fig 1 and Table 1). Undeniably, all the described outcomes bear testimony to the status of the uncovered lineages as independent evolutionary units at least for a certain period in history and thus potential independent taxonomic units.

### Correspondence to the subspecies definition

The conception and diagnosable criteria of subspecies as a taxonomic unit have gone through a long and impassioned debate [43–46]. Patten, Winker and Haig (2010) [46] figured out four essential features for subspecies from definitions proffered over years as follows: (i) no reproductive isolation between different subspecies; (ii) defining features based on genetic or developmental facts; (iii) unique breeding ranges that separate from each other for various subspecies, and (iv) diagnosable distinctions. Three points out of the four listed characteristics are indeed met by two units studied by us. First, our units were morphologically different. *M*. *h*. *himalayana* ‘apparently differs in being grayer, without the more ochraceous tints of *M*. *h*. *robusta*, and in having the black of the forehead less extensive’ as described by Howell (1929) [13]. Second, a unique breeding range (region A) for *M*. *h*. *himalayana* was found in our study, and the sampling there was sufficient to strut a compelling assessment. Though an exclusive breeding range for *M*. *h*. *robusta* was not found in the studied area; it is believed to be located somewhere else in the whole species’ range. Third, the two units were proved as not reproductively isolated. The analyses on the genetic structure revealed strong genetic differentiation accompanied by obvious admixture between the unique breeding range for the *himalayana* lineage and the mixed range of two lineages (Fig 5). Furthermore, inside the mixed range, no genetic structure was observed between the two lineages, indicating the existence of significantly frequent admixture. Correspondingly, the *F*_*st*_ values of group A vs. group B-I and group A vs. group B-II (0.0677 and 0.0765) were significantly larger than that between group B-I and group B-II (0.0067). These results serve as a sufficient demonstration for the strong gene flow between the two lineages in the mixed range and the absence of reproductive isolation between them. Last, although no defining features based on genetic or developmental facts were verified here, sufficient time has elapsed for some “heritable geographic variation in phenotype” to be built since the lineages split at about 1.03 mya, provided that the story is not too intricate. So, although a reciprocal monophyly is considered not necessary for subspecies differentiation and cannot act as a proper excuse to reject any subspecies, it does be an important positive signal indicating the occurrence of subspecies.

### Discrepancy with formerly described subspecies distribution

The subspecies rank of *M*. *h*. *himalayana* and *M*. *h*. *robusta* has been in use for years. Although no studies especially aimed at their differentiation are available, detailed descriptions of their distribution in China could be found in many taxonomic checklists, two opinions out of which will be discussed here. According to Wang (2002) [15], the nominate subspecies is distributed in Xizang, southern Xinjiang, Qinghai (Qaidam Basin) and western Gansu, while *M*. *h*. *robusta* in northwestern Yunnan, western Sichuan and Qinghai (except Qaidam Basin). However, in Smith and Xie (2009) [16], the former was thought to dwell only in southern Xizang, and the latter inhabited a pretty extensive range: Xinjiang, Xizang, Qinghai, Gansu, western Sichuan and Yunnan. Judging from our study, apparently, *M*. *h*. *himalayana* lives in a larger range than Smith and Xie (2009) [16] expected since a wide area in the northeast of Qinghai was discovered to be its unique breeding range. Also, the opinion of Wang (2002) [15] about both subspecies’ distribution in Qinghai is not so accurate because both subspecies were found outside Qaidam Basin in our study, letting alone the unique breeding range for *M*. *h*. *himalayana* outside Qaidam Basin.

### Significance of our study

Resistance and susceptibility to *Y*. *Pestis*, which can act as important determinants for the role and contribution of a particular host population in the maintenance and transmission of plague, are recognized to depend on host species and even genetic differences among individuals and populations [47–49]. Variations in plague resistance among different populations of deer mice (*Peromyscus maniculatus*) and California voles (*Microtus californicus*) were already reported, and the resistance in *M*. *californicus* was revealed to be genetic [50, 51]. Therefore, whether or not any resistance or susceptibility differences exist in subspecies level in *M*. *himalayana* is a question of future studies, which may provide constructive knowledge for the prevention and control of plague. Whatever, it is proved by our study that the genetic differentiation in the Himalaya marmot population in QTP is discrete and heterogeneous, which can serve as reliable supporting information for more precise investigations on plague epizootiology in future. Meanwhile, a unique breeding range for *M*. *h*. *himalayana* and a mixed range where both subspecies are distributed and interbreed fairly frequently are located in the northeast of QTP. If the subspecies contribute to the plague differently, corresponding prevention and control strategies should be developed for different ranges.

On the other hand, due to the key role of the Himalayan marmot in the maintenance, transmission, and prevalence of plague [4], the government always conducts extensive marmot-killing tasks. Undoubtedly, this is a potential threat for the biological diversity of the Himalayan marmot, although it is widespread and abundant in the QTP now. So this study provides scientifically based information on the subspecies differentiation for *M*. *himalayana* and can act as a guide to the government’s marmot-killing actions to control the marmot population and conserve its biological diversity simultaneously.

## Conclusions

As discussed above, the taxonomic rank of two subspecies, *M*. *h*. *himalayana* and *M*. *h*. *robusta*, was confirmed in different aspects, i.e., the well supported reciprocal monophyly, quite a long period of divergence (1.03 mya) and large genetic distance exceeding that between almost any other subspecies pair and even some species pair in the same genus, a unique breeding range for *M*. *h*. *himalayana* and clear signals for admixture between them. Provided all these proofs, we proved that the subspecies differentiation of Himalayan marmot and a unique breeding range for *M*. *h*. *himalayana*, together with a second contact range where both subspecies are distributed and interbreed, took place in the northeast of QTP.

## Supporting information

S1 File Genotypic data for all individuals at 11 microsatellite loci. (XLSX)

S1 Table Geographic information of 43 populations of *M*.*himalayana*. (DOCX)
