# Supplementary material for: Genetic evidence for subspecies differentiation of the Himalayan marmot, Marmota himalayana, in the Qinghai-Tibet Plateau
Source: PLoS One. 2017 Aug 15;12(8):e0183375. doi: 10.1371/journal.pone.0183375 (PMC5557547; doi:10.1371/journal.pone.0183375)
Supplement: S1 Table — (DOCX) [file pone.0183375.s002.docx]

**S1 Table. Geographic information of 43 populations of *M.himalayana*.**

| Pop | Code | Locality | Longitude (E) | Latitude (N) | Pop | Code | Locality | Longitude (E) | Latitude (N) |
| --- | --- | --- | --- | --- | --- | --- | --- | --- | --- |
| 1 | HZK | Kema, Huzhu | 102°16′ | 36°53′ | 23 | TDG | Gabasongduo, Tongde | 100°40′ | 35°15′ |
| 2 | LDQ | Qutan, Ledu | 102°13′ | 36°18′ | 24 | GEX | Xidatan, Geermu | 94°23′ | 35°47′ |
| 3 | MHL | Lierbao, Minhe | 102°37′ | 36°12′ | 25 | CDG | Gaduo, Chenduo | 96°40′ | 33°35′ |
| 4 | HLE | Ertang, hualong | 102°09′ | 36°07′ | 26 | CDC | Chenwen, Chenduo | 97°08′ | 33°19′ |
| 5 | XHG | Gangcha, Xunhua | 102°14′ | 35°41′ | 27 | CDZ | Zhenqin, Chenduo | 97°31′ | 33°25′ |
| 6 | TRG | Guashize, Tongren | 102°16′ | 35°29′ | 28 | CDQ | Qingshuihe, Chenduo | 97°09′ | 33°55′ |
| 7 | QLZ | Zhamashi, Qilian | 99°48′ | 38°17′ | 29 | ZDL | Lixin, Zhiduo | 96°16′ | 33°40′ |
| 8 | QLE | Ebao, Qilian | 100°49′ | 38°01′ | 30 | ZDZ | Zhiqu, Zhiduo | 95°28′ | 34°15′ |
| 9 | QLM | Mole, Qilian | 100°20′ | 37°56′ | 31 | ZDD | Duocai, Zhiduo | 95°28′ | 33°47′ |
| 10 | GCY | Yikewulan, Gangcha | 99°54′ | 37°25′ | 32 | ADA | Angsai, Zaduo | 95°47′ | 32°39′ |
| 11 | GCJ | Jiermeng, Gangcha | 99°30′ | 37°17′ | 33 | ADZ | Zhaqing, Zaduo | 95°11′ | 33°10′ |
| 12 | HYG | Ganzigou, Haiyan | 100°37′ | 37°13′ | 34 | YSZ | Zhongda, Yushu | 97°07′ | 33°00′ |
| 13 | TJX | Xinyuan, Tianjun | 99°00′ | 37°11′ | 35 | YSS | Shanglaxiu, Yushu | 96°29′ | 32°47′ |
| 14 | TJZ | Zhouqun, Tianjun | 99°21′ | 37°34′ | 36 | YSM | Xiaosumang, Yushu | 97°14′ | 32°14′ |
| 15 | TJY | Yangkang, Tianjun | 98°39′ | 37°36′ | 37 | NQD | Dongba, Nangqian | 95°34′ | 32°17′ |
| 16 | WLN | Narengou, Wulan | 98°28′ | 37°15′ | 38 | NQM | Maozhuang, Nangqian | 96°50′ | 32°22′ |
| 17 | WLY | Yamatu, Wulan | 98°03′ | 37°13′ | 39 | NQB | Baizha, Nangqian | 96°32′ | 31°58′ |
| 18 | WLW | Wulanbulang, Wulan | 98°16′ | 36°42′ | 40 | QMD | Dongfeng, Qumalai | 95°56′ | 34°04′ |
| 19 | DLQ | Qingquan, Dulan | 97°36′ | 35°52′ | 41 | QMQ | Qiuzhi, Qumalai | 95°40′ | 34°38′ |
| 20 | DHK | Keluke, Delingha | 97°05′ | 37°31′ | 42 | QMB | Bagan, Qumalai | 96°27′ | 33°52′ |
| 21 | DHH | Huaitoutala, Delingha | 96°48′ | 37°35′ | 43 | QMY | Yege, Qumalai | 95°19′ | 34°39′ |
| 22 | TDH | Hebei, Tongde | 100°49′ | 34°47′ |  |  |  |  |  |
